# Metabolic Syndrome and Combination Antiretroviral Therapy in HIV Patients in Periurban Hospital in Ghana: A Case-Control Study

**DOI:** 10.1155/2023/1566001

**Published:** 2023-02-17

**Authors:** Bartholomew Dzudzor, Samuel Essel, Latif Musah, Jennifer Adjepong Agyekum, Kwame Yeboah

**Affiliations:** ^1^Department of Medical Biochemistry, University of Ghana Medical School, Accra, Ghana; ^2^Department of Physiology, University of Ghana Medical School, Accra, Ghana; ^3^Department of Physician Assistant Studies, Central University, Accra, Ghana

## Abstract

**Background:**

There is an increasing prevalence of cardiovascular diseases (CVDs) and risk factors in HIV patients as the levels of AIDS-related mortality and morbidity decrease. Metabolic syndrome (MetS) is the accumulation of various CVD risk factors that predict the occurrence of CVDs. We investigated the prevalence of MetS and associated risk factors in HIV patients treated with combination antiretroviral therapy (cART), cART-naïve HIV patients, and non-HIV controls.

**Methods:**

In a case-control design, 158 cART-treated HIV patients, 150 cART-naïve HIV patients, and 156 non-HIV controls were recruited from a periurban hospital in Ghana. A structured questionnaire was used to collect data on demography, lifestyle, and medication. Anthropometric indices and blood pressure were measured. Fasting blood samples were collected to measure the plasma levels of glucose, lipid profile, and CD4+ cells. The presence of MetS was defined using the joint scientific statement criteria.

**Results:**

The prevalence of MetS was higher in cART-treated HIV patients compared with cART-naïve HIV patients and non-HIV controls (57.3% vs. 23.6% vs. 19.2% and *p* < 0.001, respectively). MetS was associated with cART-treated HIV patients (odds ratio (95% CI) = 7.24 (3.41–15.39) and *p* < 0.001), cART-naïve HIV patients (2.04 (1.01–4.15), *p*=0.048), and female gender (2.42 (1.39–4.23) and *p*=0.002). In cART-treated HIV patients, those on zidovudine (AZT)-based regimens were associated with increased likelihood (3.95 (1.49–10.43) and *p* < 0.006), while those on tenofovir (TDF)-based had decreased likelihood (0.32 (0.13–0.8) and *p*=0.015) of having MetS.

**Conclusion:**

In our study population, there was a high prevalence of MetS in cART-treated HIV patients compared to cART-naïve HIV patients and non-HIV controls. HIV patients on AZT-based regimens had an increased likelihood of having MetS, while those on TDF-based regimens had a reduced likelihood of having MetS.

## 1. Introduction

Sub-Saharan Africa (SSA) has the highest population of people living with HIV (PLWH) worldwide and this is associated with healthcare, socioeconomic, and developmental challenges [1]. It was reported in 2017 that 71% of the global total number of PLWH resides in SSA, with 75% of HIV-related deaths and 65% of new HIV infections occurring in this region [2]. In Ghana, the prevalence of HIV infection is 1.7% and more concentrated in urban and periurban areas [3]. The widespread availability and accessibility of combination antiretroviral therapy (cART) in low-middle-income countries have dramatically decreased the mortality attributable to HIV infection in the past decade [4]. Now, PLWHs live longer and become more susceptible to chronic diseases, especially cardiovascular diseases (CVDs) [5, 6].

The development of CVDs in patients and the general population has been associated with metabolic syndrome (MetS), which is defined as the accumulation of some cardiometabolic risk factors such as abdominal obesity, high blood pressure, dyslipidemia, and impaired fasting plasma glucose [7, 8]. The prevalence of MetS has been reported to be higher in PLWH compared to the general population [9, 10]. Even in PLWH, there is substantial variation in the prevalence of MetS, mainly due to different diagnostic criteria for MetS used across various studies, as well as differences in the sociocultural attributes of the population studied [11]. A global meta-analysis of MetS, in which only two studies from Africa were included, reported the estimated prevalence of MetS to be 29.6% [12]. A recent meta-analysis of the Sub-Saharan African population reported an estimated prevalence of MetS in PLWH to be 21.5% with substantial heterogeneity across various studies analysed [6]. In Ghana, there has been only one study that has reported the prevalence of MetS in the Ghanaian PLWH population [13].

The current treatment guidelines for HIV in Ghana require that all PLWH be treated with combination antiretroviral therapy (cART) irrespective of their disease staging and/or CD4+ lymphocyte count. The recommended first line treatment is tenofovir (TDF)-based regimen, combined with either lamivudine (3TC)/emtricitabine (FTC) or efavirenz (EFV)/nevirapine (NVP). However, a substitute for the TDF-based regime, zidovudine (ZDV)-based regimen, is widely used in managing PLWH in Ghana, especially during the past 5 years, when there was a shortage of TDF-based regimen. Protease inhibitor-based regimens are not commonly used among most PLWHs in Ghana [14]. It has been reported that these cART regimens are associated with MetS and CVDs, but these study reports are from patients in the developed countries [11, 15] and the Asian population [5]. There is no study reporting the association between cART regimens and MetS in the Ghanaian population. In this study, we compared the prevalence and associated factors of MetS in cART-treated and cART-naïve HIV patients with non-HIV participants. We also assessed the association between various cART regimens and MetS in Ghanaian PLWH.

## 2. Methods

### 2.1. Study Participants, Site, and Design

This study was a case-control design with HIV patients as cases and the controls were non-HIV individuals who visited the HIV clinic for voluntary testing of their HIV status. HIV patients were recruited by a systemic random sampling as every third consenting patient was invited to join the study, whereas non-HIV participants were recruited conveniently by invitation. HIV patients were categorized as those on cART management (cART-treated) and newly diagnosed patients who were yet to be put on cART medication (cART-naïve). The study was conducted at Atua Government Hospital, a 150-bed primary healthcare facility, located in Agormanya, a periurban town in the Eastern region of Ghana. The Agormanya area has a high prevalence of HIV infection compared to the national prevalence, and the hospital has about 1,400 HIV patients on its register. Ethical approval was obtained from the College of Health Sciences Ethical and Protocol Review Committee (CHS-Et/M.6–5.17/2018-2019), and all participants provided voluntary informed consent before joining the study.

### 2.2. Data Collection

A structured questionnaire was used to obtain data on sociodemographic factors such as age, gender, lifestyle factors (smoking and alcohol intake), medical history (hypertension, diabetes, and cardiovascular disease), current cART medication, occupation, education (school cycle completion), and marital status. Smoking status was classified as never, past (smoking cessation since more than 1 year before the survey), or current smoking, and alcohol intake was classified as drinkers and nondrinkers. The body weight and height were measured using a stadiometer in light clothing with footwear removed. The waist and hip circumferences were measured with a nonelastic tape measure parallel to the floor without compressing the skin. Pulse rate and systolic and diastolic blood pressure (average of two measures for each arm at 1-min intervals) were recorded using a semiautomatic blood pressure monitor (BF-508, Omron Healthcare, Inc., Vernon Hills, IL, USA). Hypertension was defined in the case of self-reported ongoing antihypertensive treatment and/or systolic blood pressure ≥140 mm Hg and/or diastolic blood pressure ≥90 mm Hg.

### 2.3. Biochemical Analysis

After 8–12 h of overnight fasting, approximately 5 ml of venous blood was drawn from the antecubital fossa into appropriate tubes. The samples were centrifuged at 4000 G, and the serum/plasma was aliquoted and stored at −70°C until analysis. Fasting plasma glucose (FPG), total cholesterol, high density (HDL) lipoprotein cholesterol, and plasma triglyceride levels were analysed using a biochemistry analyser (Contec BC 400, China) and commercial reagents (Randox Laboratory Reagents, UK). Low-density lipoprotein (LDL) cholesterol levels were calculated using Friedewald's formula. The CD4 cell count was measured using TriTEST reagents following an in-house, dual platform protocol, and multiset and attractors software using a FACScan flow cytometer (Becton-Dickinson, NJ, USA).

### 2.4. Definition of MetS

The definitions of MetS based on the criteria of the Joint Interim Statement (JIS), the National Cholesterol Education Program Adult Treatment Panel III (ATP III) [16], and the International Diabetes Federation (IDF) [17] were initially used. IDF definition of MetS is based on waist circumference (adjusted for Africans) >80 cm in women and >94 cm in men plus two of the following: triglycerides ≥1.7 mmol/l or specific treatment for this lipid abnormality, HDL <1 mmol/l in males and <1.3 mmol/l in females, FPG >5.6 mmol/l or previously diagnosed type 2 diabetes, systolic blood pressure ≥130 mmHg, or diastolic blood pressure ≥85 mmHg. ATP III definition is based having three or more of the following: waist circumference >88 cm in women and >102 cm in men, triglycerides ≥1.7 mmol/l, HDL <1.3 mmol/l in women or <1 mmol/l in men, FPG >6 mmol/l, or blood pressure ≥130/≥85 mmHg. JIS definition attempted to reconcile the ATP III and IDF criteria, with the criteria being similar to IDF, except that elevated waist circumference is no longer a mandatory requirement [18]. This study adopted the JIS criterion in analysis of MetS.

### 2.5. Sample Size

The minimum sample size required was computed with the online Epitools for a case-control study design. We assumed that the prevalence of MetS among non-HIV controls would be 20% and the HIV patients would have an odds ratio of 2.2 for MetS [8, 11, 13]. At the 95% significant level and 80% power, at least 128 participants were required for each group. We, therefore, targeted to recruit a minimum of 150 participants in each group for the study.

### 2.6. Data Analysis

The data were analysed using SPSS version 27. The comparisons of anthropometric indices, biochemical analytes, sociodemographic, and clinical variables were performed using ANOVA for continuous variables with normal distribution and the Kruskal–Wallis *H* test for variables with non-normal distribution. Association between categories of study participants versus MetS and its components were analysed as Pearson's *χ*^2^, with Fisher adjustment or Yate's continuity correction being appropriate. The univariate and multivariable logistic regression models were used to analyse the change in odds of MetS and HIV status, cART regimen, and clinical and sociodemographic factors. A *p* value <0.05 was considered statistically significant.

## 3. Results

### 3.1. General Characteristics of Study Participants

The mean age of the study participants was 38.4 ± 13.7 years with two-thirds being females. There was no difference in mean age among various categories of participants. There were high proportions of HIV patients who were hypertensives, underweight, and currently or formerly smoked. Compared to non-HV participants and cART-naïve HIV patients, cART-treated HIV patients had higher waist circumference, waist-hip ratio, percentage body fat, diastolic and mean blood pressure, and heart rate. The cART-treated HIV patients had higher levels of FPG, triglycerides, and total and LDL cholesterol compared with non-HIV participants (Table 1).

### 3.2. Prevalence of Metabolic Syndrome and Its Components

**Figure 1:**
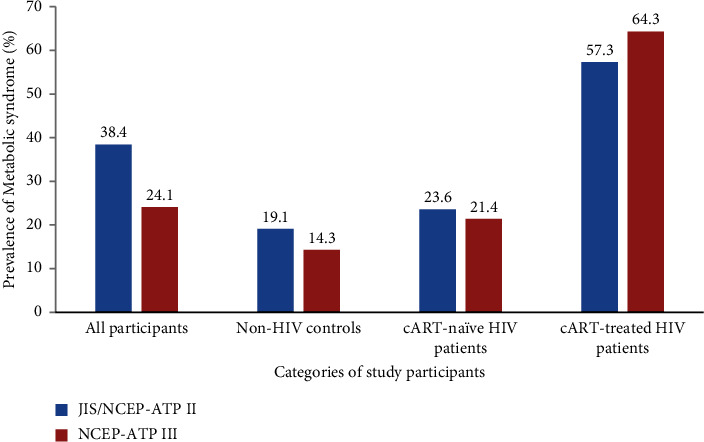
Prevalence of metabolic syndrome among study participants. The prevalence of MetS was higher in the cART-treated HIV patients compared to the cART-naïve or non-HIV controls (*p* < 0.001). There was no difference in the prevalence of MetS between cART-naïve HIV patients and non-HIV controls (*p*=0.21).

The prevalence of MetS was similar using the NCEP-ATP III and JIS criteria. However, compared to the JIS and NCEP-ATP III criteria, the prevalence of MetS was higher using the IDF criterion except in cART-treated HIV patients. Irrespective of the criteria used, the prevalence of MetS was higher in cART-treated HIV patients compared to cART-naïve HIV patients and non-HIV participants. There was no difference in the prevalence of MetS between cART-nave HIV patients and non-HIV participants (Figure 1). Subsequently, we chose to use the JIS criterion to define MetS in the analyses. Concerning the individual components of MetS, IFG, abdominal obesity, low HDL cholesterol, and hypertriglyceridemia were associated with various categories of study participants (Table 2). In logistic regression analyses, compared to non-HIV controls, cART-treated HIV patients had increased odds of having IFG, low HDL cholesterol, hypertriglyceridemia, and MetS in both unadjusted and adjusted models (Table 3).

### 3.3. Factors Associated with Metabolic Syndrome

Among the entire study participants, age, being cART-treated HIV patient, females, and being self-employed were associated with increased odds of having MetS, whereas having formal education upto senior high school and tertiary was associated with decreased odds of having MetS in unadjusted logistic regression models. In the adjusted logistic regression models, being cART-treated or cART-naïve HIV patients and female gender were associated with increased odds of having MetS (Table 4).

### 3.4. Association between Metabolic Syndrome and cART Treatment

The average duration of HIV infection in cART-treated HIV patients was 7.6 ± 4.6 years, and the average duration of cART treatment was 7.2 ± 4.5 years. For the cART medication regimen, 94 (59.5%) patients were treated with TDF/3TC/NVP or EFV regimens, 52 (32.9%) patients were on AZT/3TC/NVP or EFV regimens, and 12 (7.6%) patients were on LPV/r-based regimens. Patients on the AZT-based regimens had increased odds of having MetS, while those on the TDF-based regimen had decreased odds of having MetS in both unadjusted and adjusted logistic regression models. Patients on the EFV-based regimen had decreased odds of MetS in an unadjusted regression model but not in the adjusted model. There was no association between MetS and patients on NVP-based or LVP/r-based regimens (Table 5). The association between MetS and the components of MetS is shown in Table S1 (Supplementary digital content, online only).

## 4. Discussion

### 4.1. Major Findings

The findings of this study indicate that cART-treated HIV patients had a higher prevalence of MetS compared to cART-naïve HIV patients and non-HIV controls, but no difference in the prevalence of MetS between cART-naïve HIV patients and non-HIV controls. MetS was associated with HIV status and female gender in all study participants. In cART-treated patients, being on the AZT-based regimen was associated with increased odds of having MetS and being on the TDF-based regimen was associated with decreased odds of having MetS.

### 4.2. Prevalence of MetS

The prevalence of MetS in our study population is similar to a previous study in Ghana, which reported the prevalence of MetS to be 25.2% in cART-naïve HIV patients and 50.3% in cART-treated HIV patients using the NCEP-ATP III criterion [13]. However, this study did not include non-HIV controls, and no multivariable analysis was performed to determine the predictors of MetS and the effects of cART regimens. Other studies in the Sub-Saharan African population have reported the prevalence of MetS similar to what was found in our study population. In the Cameroonian population, Ngatchou et al. reported the prevalence of MetS in HIV patients to be 47%, compared to 21% in non-HIV controls using the IDF criterion [19]. A similar finding of a high prevalence of MetS (58%) was reported by Muyanga et al. in Ugandan cART-treated HIV patients [20]. However, other studies conducted in Sub-Saharan Africa reported a lower prevalence of MetS compared to what was found in our study. For example, in Nigeria, the prevalence of MetS in PLWH was reported to be 12.7%, 17.2%, and 21% by ATP III, IDF, and JIS criteria [21]. Similarly, the reports from studies conducted in Ivory Coast, Burkina Faso, and South Africa have reported the prevalence of MetS in the cART-treated HIV patients to be 6.2%, 18%, and 8.7%, respectively [7, 9, 10]. The variations in the prevalence of MetS may be attributed to variations in the criteria used to define MetS, geocultural differences in the studied populations, and various levels of exposure to CVDs [11, 16]. In addition to HIV infection and cART treatment that were considered to be exposure variables of MetS, other factors such as diet, physical inactivity, stress, urbanization, and epigenetics may contribute to the development and variation in the prevalence of MetS [17]. Unfortunately, we did not measure these factors in this current study.

Concerning the components of MetS, impaired fasting glucose, low HDL cholesterol, and hypertriglyceridemia were more common in the cART-treated HIV patients compared to cART-naïve HIV patients or non-HIV controls after adjusting for confounders. This observation of glucose and lipid abnormalities is consistent with previous studies among HIV population in Ethiopia [8, 22], Zambia [23], and Cameroon [24]. The HIV accessory protein, viral protein R, and cART combination have been reported to affect insulin sensitivity by regulating hepatic lipid metabolism, leading to hyperglycemia and dyslipidemia [25]. We did not find any association between MetS and lifestyle factors such as alcohol intake and cigarette smoking. There were conflicting reports on the role of alcohol intake on MetS in HIV patients.

In this study, we employed the quantity/frequency approach to measure alcohol exposure within the past year. However, most of the drinkers in our study population, as is the case of most rural and periurban populations in Africa, consumed locally prepared alcoholic beverage without any standard serving volume (example of some response from participants: calabash full and half of medium gourd bottle) and varying ethanol content based on the preparation procedure (e.g., a day/week fermented palm wine and 4-day fermented malt drink). Therefore, we could not quantify the amount of ethanol exposure. We ended up categorizing them as “drinkers” and “nondrinkers.” The “nondrinkers” are lifetime abstainers, who have no recollection of alcohol intoxication, and those classified as “drinkers” took more than enough alcohol (intoxicated) at least once a month. This method of measuring alcohol exposure has masked the role of alcohol consumption and metabolic syndrome in our study. There are conflicting reports on the relationship between alcohol intake and MetS. Meta-analysis indicates the high prevalence of MetS in people with alcohol use disorders [26], but lower prevalence of MetS was found in those with low and moderate alcohol intake [27, 28]. In addition, the timing of drinking alcohol vis-à-vis meal intake may have dramatic effect on the metabolic effect of alcohol [29]. Similar to our findings, some studies reported no association between smoking and MetS [30, 31], whereas others reported an association between these parameters and MetS [7, 10]. It should be noted that our study population has few numbers of smokers to draw any reasonable conclusion on the relationship between smoking and MetS.

### 4.3. MetS and cART Regimen

Considering the cART-treated HIV patients, those on the AZT-based regimens had an increased likelihood of having MetS, while those on the TDF-based regimens had decreased likelihood of having MetS. This observation agrees with the findings of Labhardt et al. who reported that, in South African HIV patients, those on AZT-based regimens had increased likelihood of MetS compared to those on TDF-based regimens [32]. In contrast to our findings, Sashindran and Singh reported that TDF-based regimens rather increase the likelihood of MetS in Indian HIV patients [5]. The AZT-based regimen has been shown to cause oxidative damage, a major mechanistic pathway of MetS and CVDs, through the induction of mitochondrial dysfunction by inhibition of DNA polymerase-*γ* activity [33]. Moreover, a metabolomic study showed that the pathway through which the AZT-based regimen leads to oxidative stress is by altering the metabolism of glutamine, glutamate, glutathione, and arginine biosynthesis as well as alanine, aspartate, and glutamate metabolism [11]. Fortunately, it has been demonstrated in a meta-analysis that the TDF-based regimen has better viral suppression and tolerability compared to the AZT-based regimen [34, 35].

### 4.4. Limitations of the Study

The major limitation of our study is that it was conducted in a single periurban health facility, limiting the generalization of our findings to the entire Ghanaian population. Furthermore, we collected data cross-sectionally, and hence, we cannot infer causality from our results. We cannot be specific that HIV infection and/or cART treatment resulted in increased MetS in our study population because it may be possible that some of our study participants had MetS before HIV infection and/or cART treatment. Moreover, we did not measure some inflammatory markers which may have explained the possible mechanisms underlining the high levels of MetS observed in the patient groups. We recommend that future studies should utilize a multicentre longitudinal design to investigate the possible mechanisms underlining the high burden of MetS in HIV infection and cART treatment.

## 5. Conclusion

In our study population, the prevalence of MetS is high in the cART-treated HIV patients compared to the cART-naïve HIV patients and non-HIV controls. HIV patients on AZT-based regimens had an increased likelihood of having MetS, while those on TDF-based regimens had a low likelihood of having MetS. Future studies may use a longitudinal study design to monitor the development and management of MetS from HIV infection and initiation of cART treatment.

## Figures and Tables

**Table 1:** General characteristics of study participants.

	All participants	Non-HIV controls	ART-naïve HIV patients	cART-treated HIV patients	*p*
*N*	464	156	150	158	
Age, years	38.4 ± 13.7	36.7 ± 14.4	38.2 ± 11.6	39 ± 11.4	0.109
Females, *n* (%)	312 (67.2)	106 (67.9)	84 (56)	122 (77.2)	0.02
Married, *n* (%)	198 (42.7)	70 (44.9)	62 (41.3)	66 (41.8)	0.79
Smoking, *n* (%)					0.029
Current	16 (3.4)	2 (1.3)	4 (2.7)	10 (6.3)	
Former	57 (15.9)	9 (5.8)	22 (14.6)	26 (16.5)	
Never	187 (80.6)	71 (92.9)	124 (82.7)	132 (83.5)	
Alcohol intake, *n* (%)	102 (22)	38 (24.4)	36 (24)	28 (17.7)	0.55
Waist circumference (cm)	87 ± 12	85 ± 11	84 ± 11	90 ± 12^#^^*∗*^	0.002
Hip circumference (cm)	102 ± 11	103 ± 11	99 ± 11^*∗*^	103 ± 11	0.074
Body height (cm)	164 ± 7	164 ± 8	164 ± 7	162 ± 7	0.194
Waist-hip ratio	0.85 ± 0.08	0.82 ± 0.08	0.84 ± 0.07^*∗*^	0.88 ± 0.08^*∗*^^#^	<0.001
Body weight (kg)	60 ± 13.2	68 ± 13.3	64.4 ± 7.3	65.8 ± 14	0.204
BMI (kg/m^2^)	24.8 ± 5	25.4 ± 4.7	23.7 ± 4.4^*∗*^	25.3 ± 5.7	0.061
BMI categories, *n* (%)					0.024
Underweight	34 (7.4)	6 (3.9)	16 (10.7)	12 (7.6)	
Normal	238 (51.5)	70 (45.5)	94 (62.7)	74 (46.8)	
Overweight	116 (25.1)	46 (29.9)	18 (12)	52 (32.9)	
Obese	74 (16)	32 (20.8)	22 (14.7)	20 (12.7)	
Body fat (%)	31 ± 12.2	32.4 ± 12.5	27.6 ± 11.8^*∗*^	32.8 ± 11.6^*∗*^^#^	0.014
Systolic BP (mmHg)	134 ± 18	132 ± 13	133 ± 19	137 ± 22	0.184
Diastolic BP (mmHg)	83 ± 11	80 ± 9	83 ± 12	86 ± 12^*∗*^^#^	0.008
Mean BP (mmHg)	100 ± 14	98 ± 10	99 ± 14	104 ± 16^*∗*^^#^	0.007
Pulse BP (mmHg)	51 ± 13	52 ± 10	50 ± 10	51 ± 13	0.672
Heart rate (bpm)	74 ± 9	72 ± 8	73 ± 9	80 ± 8^*∗*^^#^	<0.001
Hypertension, *n* (%)	162 (34.9)	48 (30.8)	46 (30.7)	68 (43)	0.031
FPG (mmol/l)	5.2 ± 0.8	5 ± 0.9	5.1 ± 0.8	5.6 ± 0.8^*∗*^	<0.001
Triglycerides (mmol/l)	1.4 ± 0.4	1.4 ± 0.3	1.4 ± 0.4	1.6 ± 0.4^*∗*^	<0.001
Total cholesterol (mmol/l)	5.1 ± 1.2	4.8 ± 1.2	5 ± 1.1	5.6 ± 1.1^*∗*^	<0.001
HDL cholesterol (mmol/l)	1.5 ± 0.4	1.6 ± 0.4	1.4 ± 0.4	1.5 ± 0.5	0.128
LDL cholesterol (mmol/l)	3 ± 0.9	2.6 ± 0.9	3 ± 0.8^*∗*^	3.3 ± 0.7^*∗*^^#^	<0.001
Current CD4 count (cells/mm^2^)	405 (273–562)		430 (327–534)	403 (253–583)	0.804

^*∗*^: *p* < 0.05 compared to non-HIV; ^#^: *p* <  0.05 compared to treatment naïve HIV patients.

**Table 2:** Prevalence of components of metabolic syndrome among study participants by HIV status.

	Non-HIV controls, *n* (%)	HAART-naïve HIV patients, *n* (%)	HAART-treated HIV patients, *n* (%)	*p*
IFG	54 (34.6)	54 (36)	100 (63.3)	<0.001
High systolic BP	84 (53.8)	84 (54.7)	88 (55.7)	0.947
Abdominal obesity	88 (56.4)	68 (45.3)	112 (70.9)	<0.001
Low HDL	36 (23.1)	40 (26.7)	62 (39.2)	0.004
Hypertriglyceridemia	28 (17.9)	30 (20)	82 (51.9)	<0.001

IFG, impaired fasting plasma glucose; BP, blood pressure; HDL, high density lipoprotein cholesterol.

**Table 3:** Association between metabolic syndrome and its components among study participants.

	ART-naïve HIV patients, OR (95% CI)		cART-treated HIV patients, OR (95% CI)	
	Unadjusted	Adjusted	Unadjusted	Adjusted
IFG	1.06 (0.67–1.7)	0.85 (0.49–1.48)	3.26 (2.05–5.17)	2.19 (1.19–4.03)
High systolic BP	1.03 (0.66–1.62)	0.96 (0.55–1.69)	1.08 (0.69–1.68)	0.56 (0.29–1.07)
Abdominal obesity	0.64 (0.41–1.01)	0.52 (0.29–0.97)	1.88 (1.18–3)	0.8 (0.39–1.65)
Low HDL	1.21 (0.72–2.04)	2.17 (1.14–4.12)	2.15 (1.32–3.52)	6.22 (2.91–13.32)
Hypertriglyceridemia	1.14 (0.65–2.03)	1.07 (0.53–2.16)	4.93 (2.95–8.25)	4.72 (2.23–9.96)
MetS	1.4 (0.83–2.35)	1.3 (0.69–2.46)	6.54 (3.96–10.78)	4.62 (2.35–9.07)

The model for adjusted OR were adjusted for age, gender, marital status, alcohol and smoking status, employment, educational level, and BMI. IFG, impaired fasting plasma glucose; BP, blood pressure; HDL, high density lipoprotein cholesterol; MetS, metabolic syndrome.

**Table 4:** Determinants of metabolic syndrome in all study participants.

	MetS (OR (95% CI))		*P*
	Unadjusted	Adjusted	
*HIV status: reference* *=* *non-HIV*			
ART-naïve	1.4 (0.83–2.35)	2.04 (1.01–4.15)	0.048
cART-treated	6.54 (4–10.78)	7.24 (3.41–15.39)	<0.001

Age	1.03 (1.02–1.05)	1.01 (0.99–1.02)	0.657
Females	2.98 (1.91–4.63)	2.89 (1.71–4.87)	<0.001
Unmarried	0.71 (0.49–1.03)	0.77 (0.47–1.26)	0.298
Alcohol intake	0.68 (0.42–1.08)	0.93 (0.53–1.62)	0.79
Current smoking	1.64 (0.6–4.44)	1.05 (0.34–3.22)	0.931

*Employment (reference: unemployed)*			
Employed	1.01 (0.54–1.87)	1.12 (0.55–2.25)	0.762
Self-employed	2.23 (1.17–4.26)	1.36 (0.64–2.86)	0.422

*Education: reference* *=* *no formal education*			
Basic/JHS	0.79 (0.42–1.46)	1.67 (0.8–3.51)	0.175
SHS/Tech	0.24 (0.11–0.53)	0.79 (0.3–2.08)	0.63
Tertiary	0.22 (0.11–0.44)	1.05 (0.4–2.77)	0.927

MetS, metabolic syndrome; cART, combination antiretroviral therapy; JHS, junior high school; SHS, senior high school; Tech, technical/vocational education.

**Table 5:** Association between metabolic syndrome and cART regimen in cART-treated HIV patients.

	MetS		Unadjusted OR (95% CI)	*p*	Adjusted OR (95% CI)^*∗*^	*p*
	Absent	Present				
TDF-based	42 (75)	58 (56.9)	0.44 (0.21–0.9)	0.025	0.41 (0.18–0.98)	0.044
AZT-based	12 (21.4)	44 (43.1)	2.78 (1.32–5.88)	0.007	2.63 (1.11–6.2)	0.028
EFV-based	42 (75)	60 (58.8)	0.48 (0.23–0.98)	0.044	0.51 (0.21–1.2)	0.508
NVP-based	12 (21.4)	34 (33.3)	1.83 (0.86–3.92)	0.118	1.83 (0.75–4.46)	0.182
LPV/r-based	4 (7.1)	8 (7.8)	1.62 (0.46–5.74)	0.458	1.5 (0.27–8.31)	0.64

^*∗*^Adjusted for age, gender, marital status, alcohol and smoking status, employment, educational level. and CD4 cell level. MetS, metabolic syndrome; AZT, zidovudine; EVF efavirenz; LPV/r, Lopinavir/ritonavir; NVP, nevirapine; TDF, tenofovir. Lamivudine was part of the medication regimen for 154 (97.5%) patients, and hence, we did not analyse it. Stavudine and emtricitabine were taken by 2 and 4 patients, respectively, and hence, we did not include them in our analyses.

## Data Availability

The dataset supporting the conclusions of this study is available and can be requested from the corresponding author.

## Abbreviations:

AZT: Zidovudine
CVDs: Cardiovascular diseases
EVF: Efavirenz
cART: Combination antiretroviral therapy
HIV: Human immunodeficiency virus
MetS: Metabolic syndrome
NVP: Nevirapine
PLWHI: People living with HIV
SSA: Sub-Saharan Africa
TDF: Tenofovir.

## Supplementary Materials

Supplementary Materials The Table S1 shows the association between the various components of metabolic syndrome and cART regimens from unadjusted and adjusted logistic regression models in cART-treated HIV patients. The results showed that the TDF-based regimen was associated with decreased odds of having low HDL cholesterol, while the AZT-based regimen was associated with increased odds of having low HDL cholesterol levels in both unadjusted and adjusted models. These results are consistent with previous findings that TDF-based regimens were associated with 20% increase in HDL cholesterol levels in 48 weeks [36] and substituting TDF in cART with tenofovir alafenamide was associated with decrease in HDL cholesterol [37]. In addition, in Malawian HIV patients, it was reported that those with reduced HDL cholesterol in relation to total plasma cholesterol had significant increased mortality [38].
