# Supplementary material for: Metabolic Syndrome and Combination Antiretroviral Therapy in HIV Patients in Periurban Hospital in Ghana: A Case-Control Study
Source: AIDS Res Treat. 2023 Feb 17;2023:1566001. doi: 10.1155/2023/1566001 (PMC9957619; doi:10.1155/2023/1566001)
Supplement: Supplementary Materials — The Table S1 shows the association between the various components of metabolic syndrome and cART regimens from unadjusted and adjusted logistic regression models in cART-treated HIV patients. The results showed that the TDF-based regimen was associated with decreased odds of having low HDL cholesterol, while the AZT-based regimen was associated with increased odds of having low HDL cholesterol levels in both unadjusted and adjusted models. These results are consistent with previous findings that TDF-based regimens were associated with 20% increase in HDL cholesterol levels in 48 weeks [36] and substituting TDF in cART with tenofovir alafenamide was associated with decrease in HDL cholesterol [37]. In addition, in Malawian HIV patients, it was reported that those with reduced HDL cholesterol in relation to total plasma cholesterol had significant increased mortality [38]. [file 1566001.f1.docx]

**Supplementary digital content**

Table S1 Association between cART regimen and components of MetS in cART-treated HIV patients.

| *Impaired fasting glucose* | | | Unadjusted OR (95% CI) | p | Adjusted OR (95% CI) | p |
| --- | --- | --- | --- | --- | --- | --- |
|  | Absent | Present |  |  |  |  |
| TDF-based | 34 (58.6) | 66 (66) | 1.37 (0.7 – 2.67) | 0.354 | 1.92 (0.85 – 4.31) | 0.115 |
| AZT-based | 22 (37.9) | 34 (34) | 0.84 (0.43 – 1.65) | 0.619 | 0.55 (0.25 – 1.24) | 0.149 |
| EFV-based | 44 (75.9) | 58 (58) | **0.44 (0.21 – 0.9)** | **0.025** | 0.48 (0.2 – 1.17) | 0.107 |
| NVP-based | 12 (20.7) | 34 (34) | 1.97 (0.93 – 4.21) | 0.079 | 1.66 (0.68 – 4.05) | 0.269 |
| LPV/r-based | 2 (3.4) | 8 (8) | 2.43 (0.5 – 11.88) | 0.271 | 2.8 (0.41 – 18.89) | 0.292 |
| *High systolic blood pressure* | | |  |  |  |  |
| TDF-based | 46 (65.7) | 54 (61.4) | 0.83 (0.43 – 1.59) | 0.573 | 0.64 (0.19 – 2.1) | 0.459 |
| AZT-based | 22 (31.4) | 34 (38.6) | 1.37 (0.71 – 2.66) | 0.347 | 1.7 (0.51 – 5.68) | 0.385 |
| EFV-based | 48 (68.6) | 54 (61.4) | 0.73 (0.38 – 1.41) | 0.347 | 0.89 (0.28 – 2.82) | 0.846 |
| NVP-based | 18 (25.7) | 28 (31.8) | 1.35 (0.67 – 2.71) | 0.402 | 1.92 (0.61 – 6.03) | 0.264 |
| LPV/r-based | 4 (5.7) | 6 (6.8) | 1.21 (0.33 – 4.46) | 0.777 | 0.12 (0.01 – 1.29) | 0.08 |
| *Abdominal obesity* | | |  |  |  |  |
| TDF-based | 32 (69.6) | 68 (60.7) | 0.68 (0.33 – 1.41) | 0.296 | 0.81 (0.31 – 2.14) | 0.674 |
| AZT-based | 14 (30.4) | 42 (37.5) | 1.37 (0.68 – 2.86) | 0.4 | 0.83 (0.32 – 2.13) | 0.699 |
| EFV-based | 34 (73.9) | 68 (60.7) | 0.55 (0.26 – 1.17) | 0.118 | 0.56 (0.2 – 1.58) | 0.273 |
| NVP-based | 10 (21.7) | 36 (32.1) | 1.71 (0.76 – 3.81) | 0.194 | 1.92 (0.68 – 5.39) | 0.218 |
| LPV/r-based | 2 (4.3) | 8 (7.1) | 1.69 (0.35 – 8.29) | 0.516 | 0.7 (0.09 – 5.3) | 0.727 |
| *Low HDL cholesterol* | | |  |  |  |  |
| TDF-based | 68 (70.8) | 32 (51.6) | **0.44 (0.22 – 0.85)** | **0.015** | **0.38 (0.17 – 0.87)** | **0.022** |
| AZT-based | 28 (29.2) | 28 (45.2) | **2 (1.03 – 3.89)** | **0.041** | **2.48 (1.09 – 5.64)** | **0.031** |
| EFV-based | 62 (64.6) | 40 (64.5) | 1 (0.51 – 1.94) | 0.993 | 0.98 (0.42 – 2.28) | 0.959 |
| NVP-based | 28 (29.2) | 18 (29) | 0.99 (0.49 – 2.01) | 0.986 | 0.94 (0.39 – 2.23) | 0.88 |
| LPV/r-based | 6 (6.3) | 4 (6.5) | 1.03 (0.28 – 3.82) | 0.959 | 1.66 (0.22 – 12.66) | 0.627 |
| *Hypertriglyceridemia* | | |  |  |  |  |
| TDF-based | 48 (63.2) | 52 (63.4) | 1.01 (0.53 – 1.93) | 0.973 | 1.07 (0.49 – 2.35) | 0.857 |
| AZT-based | 26 (34.2) | 30 (36.6) | 1.11 (0.58 – 2.13) | 0.755 | 0.96 (0.44 – 2.1) | 0.923 |
| EFV-based | 48 (63.2) | 54 (65.9) | 1.12 (0.59 – 2.16) | 0.723 | 1.5 (0.65 – 3.37) | 0.352 |
| NVP-based | 24 (31.6) | 22 (26.8) | 0.79 (0.4 – 1.58) | 0.512 | 0.63 (0.27 – 1.47) | 0.283 |
| LPV/r-based | 4 (5.3) | 6 (7.3) | 1.42 (0.39 – 5.24) | 0.598 | 1.27 (0.21 – 7.8) | 0.799 |

Adjusted for age, gender, marital status, alcohol and smoking status, employment, educational level and CD4 cells levels.

The Table S1 shows the association between the various components of metabolic syndrome and cART regimens from unadjusted and adjusted logistic regression models in cART-treated HIV patients. The results showed that TDF-based regimen was associated with decreased odds of having low HDL cholesterol, while AZT-based regimen was associated with increased odds of having low HDL cholesterol levels in both unadjusted and adjusted models. This results is consistent with previous findings that TDF-based regimens were associated with 20% increase in HDL cholesterol levels in 48 weeks [36] and substituting TDF in cART with tenofovir alafenamide was associated with decrease in HDL cholesterol [37]. In additon, in Malawian HIV patients, it was reported that those with reduced HDL cholesterol in relation to total plasma cholesterol had significant increased mortality [38].
